# Role of Key *TYMS* Polymorphisms on Methotrexate Therapeutic Outcome in Portuguese Rheumatoid Arthritis Patients

**DOI:** 10.1371/journal.pone.0108165

**Published:** 2014-10-03

**Authors:** Aurea Lima, Vítor Seabra, Miguel Bernardes, Rita Azevedo, Hugo Sousa, Rui Medeiros

**Affiliations:** 1 CESPU, Institute of Research and Advanced Training in Health Sciences and Technologies, Department of Pharmaceutical Sciences, Higher Institute of Health Sciences-North (ISCS-N), Gandra PRD, Portugal; 2 Molecular Oncology Group CI, Portuguese Institute of Oncology of Porto (IPO-Porto), Porto, Portugal; 3 Abel Salazar Institute for the Biomedical Sciences (ICBAS) of University of Porto, Porto, Portugal; 4 Faculty of Medicine of University of Porto (FMUP), Porto, Portugal; 5 Rheumatology Department of São João Hospital Center, Porto, Portugal; 6 Virology Service, Portuguese Institute of Oncology of Porto (IPO-Porto), Porto, Portugal; 7 Research Department-Portuguese League Against Cancer (LPCC-NRNorte), Porto, Portugal; National Institute for Viral Disease Control and Prevention, CDC, China, China

## Abstract

**Background:**

Therapeutic outcome of rheumatoid arthritis (RA) patients treated with methotrexate (MTX) can be modulated by thymidylate synthase (TS) levels, which may be altered by genetic polymorphisms in TS gene (*TYMS*). This study aims to elucidate the influence of *TYMS* polymorphisms in MTX therapeutic outcome (regarding both clinical response and toxicity) in Portuguese RA patients.

**Methods:**

Clinicopathological data from 233 Caucasian RA patients treated with MTX were collected, outcomes were defined and patients were genotyped for the following *TYMS* polymorphisms: 1) 28 base pairs (bp) variable number tandem repeat (rs34743033); 2) single nucleotide polymorphism C>G (rs2853542); and 3) 6 bp sequence deletion (1494del6, rs34489327). Chi-square and binary logistic regression analyses were performed, using genotype and haplotype-based approaches.

**Results:**

Considering *TYMS* genotypes, 3R3R (*p* = 0.005, OR = 2.34), 3RC3RG (*p* = 0.016, OR = 3.52) and 6bp− carriers (*p* = 0.011, OR = 1.96) were associated with non-response to MTX. Multivariate analysis confirmed the increased risk for non-response to MTX in 6bp− carriers (*p* = 0.016, OR = 2.74). Data demonstrated that *TYMS* polymorphisms were in linkage disequilibrium (*p*<0.00001). Haplotype multivariate analysis revealed that haplotypes harboring both 3R and 6bp− alleles were associated with non-response to MTX. Regarding MTX-related toxicity, no statistically significant differences were observed in relation to *TYMS* genotypes and haplotypes.

**Conclusion:**

Our study reveals that *TYMS* polymorphisms could be important to help predicting clinical response to MTX in RA patients. Despite the potential of these findings, translation into clinical practice needs larger studies to confirm these evidences.

## Introduction

**Figure 1:**
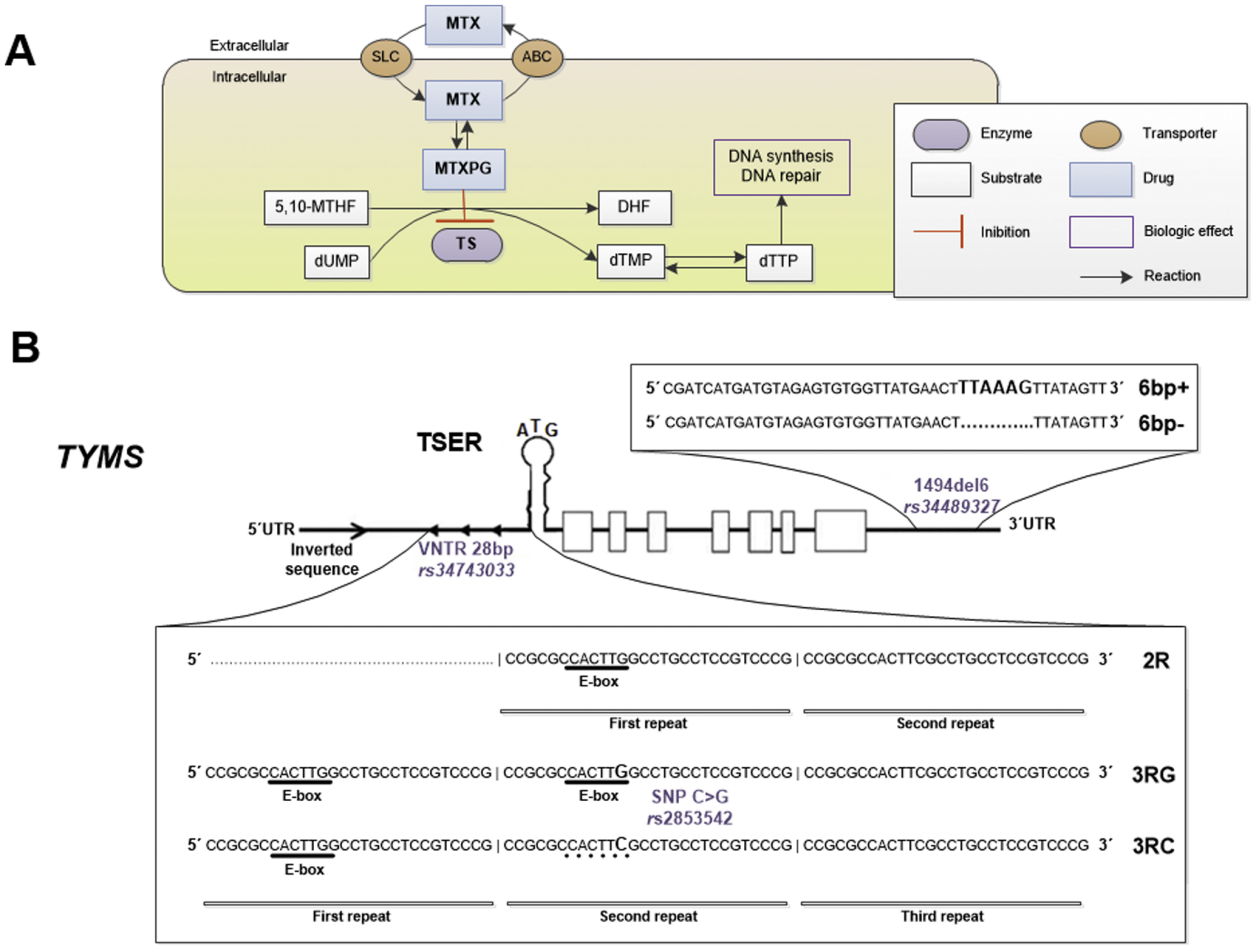
Part of MTX action mechanism in which thymidylate synthase (TS) is involved (A). MTX enters the cell after binding to folate transporters, mainly by solute carriers (SLC), and can be exported by members of the ATP-binding cassette (ABC) transporters family. To prevent MTX rapid efflux from cells and enhance its intracellular retention, MTX is polyglutamated by the enzyme folylpolyglutamyl synthase into MTX polyglutamates (MTXPGs) which inhibit TS activity. TS is a key protein for the *de novo* pyrimidine synthesis and is responsible for the simultaneous conversion of deoxyuridine monophosphate (dUMP) and 5,10-methylenetetrahydrofolate (5,10-MTHF) to deoxythymidine monophosphate (dTMP) and dihydrofolate (DHF). Subsequently, the dTMP is phosphorylated to deoxythymidine triphosphate (dTTP) and used for the DNA synthesis and repair. *TYMS* structure and location of VNTR 28 bp (rs34743033), SNP C>G (rs2853542) and 1494del6 (rs34489327) polymorphisms (B). 5,10-MTHF: 5,10-methylenetetrahydrofolate; A: adenine; ABC: ATP-binding cassette; bp: base pairs; C: cytosine; del: deletion; DHF: dihydrofolate; dTMP: deoxythymidine monophosphate; dTTP: deoxythymidine triphosphate; dUMP: deoxyuridine monophosphate; E-box: enhancer box; G: guanine; MTXPG: methotrexate polyglutamates; R: repeat; SLC: solute carrier; SNP: single nucleotide polymorphism; TS: thymidylate synthase (protein); TSER: thymidylate synthase enhancer region; T: thymine; *TYMS*: thymidylate synthase (gene); UTR: untranslated region; VNTR: variable number tandem repeat.

Methotrexate (MTX) is the cornerstone for rheumatoid arthritis (RA) treatment and is the most widely used disease-modifying antirheumatic drug (DMARD) in newly diagnosed patients [1], [2]. Despite its cost-effectiveness, therapeutic outcome is variable mainly concerning to MTX clinical response and/or development of MTX-related toxicity [3]–[7]. MTX is an antifolate drug with important anti-inflammatory and antiproliferative effects, partly achieved by the intracellular inhibition of thymidylate synthase (TS) [8]–[10]. TS is a key protein for the *de novo* pyrimidine synthesis and is responsible for the simultaneous conversion of deoxyuridine monophosphate (dUMP) and 5,10-methylenetetrahydrofolate (5,10-MTHF) to deoxythymidine monophosphate (dTMP) and dihydrofolate (DHF). Subsequently, the dTMP is phosphorylated to deoxythymidine triphosphate (dTTP) and used for the deoxyribonucleic acid (DNA) synthesis and repair [6], [11], [12] (Figure 1A). Since TS levels were found to be predictive of MTX therapeutic outcome [13], [14] and genetic polymorphisms in TS gene (*TYMS*) have been associated with TS levels [15], [16], pharmacogenomics has raised great interest and, in fact, some studies have attempted to clarify the influence of genetic variations on clinical response to MTX in RA [17]. The most studied polymorphisms (rs34743033, rs2853542 and rs34489327) are represented on Figure 1B. Polymorphism rs34743033 is a 28 base pairs (bp) variable number tandem repeat (VNTR), located on 5′ untranslated region (UTR) [18]. Is characterized by exhibiting a putative Enhancer box (E-box) sequence on the first 28 bp repeat of 2R allele and on the two first repeats of 3R allele [15], [19]. Therefore, a higher number of repeats should increase the amount of E-box binding sites for the upstream stimulating factors (USF), leading to an increased transcription of *TYMS* and, consequently, to higher TS levels [20]. In addition, a single nucleotide polymorphism (SNP) characterized by a cytosine to guanine (C>G) transition on the twelfth nucleotide of the second repeat of VNTR 3R allele (rs2853542) has been described [15]. In the presence of cytosine (3RC) the E-box seems to be disrupted, reducing the stimulation of transcription in comparison to 3RG, thereby decreasing TS levels [15]. Since this SNP occurs within the *TYMS* 28 bp VNTR polymorphism, several studies have been performed combining the information from both *TYMS* enhancer region (TSER) polymorphisms [6], [21]. Another important polymorphism is a 6 bp sequence (TTAAAG) deletion (1494del6, rs34489327) at 3′UTR, which seems to affect a region of TS pre-messenger ribonucleic acid (mRNA) that contains *cis* adenylate-uridylate-rich elements (AREs) [22], [23]. These elements bind to a *trans* AU-rich factor 1 (AUF1), preferentially in the presence of deletion allele (6bp−), diminishing mRNA stability and, consequently, decreasing TS levels [16], [22], [23]. Therefore, the aim of this study was to elucidate the clinical relevance of these *TYMS* polymorphisms, by genotype and haplotype-based approaches, in MTX therapeutic outcome of Portuguese RA patients.

## Methods

### Patients and study design

A retrospective study was performed between January 2009 and December 2012 at São João Hospital Center (Porto, Portugal) in a cohort of consecutive Caucasian patients (≥18 years) with RA treated with MTX. Patients were excluded from the study if there was history of drug abuse, recent pregnancy or desire to become pregnant. The study was approved by the Ethical Committee of São João Hospital Center (reference 33/2009), procedures were considered to be according to the standards of the Helsinki Declaration and all patients provided an informed written consent.

After diagnosis, patients were classified according the 1987 criteria of the American College of Rheumatology (ACR) and reclassified according the 2010 criteria of the ACR and the European League Against Rheumatism (EULAR) [24]. All patients were initially treated with 10 mg *per os* (PO)/week of MTX in monotherapy. This dose was increased 5 mg at each three weeks if the patients did not meet the EULAR criteria for response, i.e., if presented a Disease Activity Score in 28 joints (DAS28) >3.2. Every 3 months treatment response was evaluated and, on the: 1) first evaluation, if patients have no response or show gastrointestinal toxicity, administration route was changed to subcutaneous (SC); 2) second evaluation, if maximum tolerable dose was used without response, MTX therapy was discontinued or associated with other synthetic DMARD; and 3) third evaluation, in patients without response and other contraindication, therapy was changed by associating a biological DMARD. The occurrence of MTX-related toxicity was registered at each visit and, according to severity, MTX dose was adjusted or discontinued. Folic acid supplementation was prescribed to all patients for the prevention of toxicity occurrence and their regular compliance was registered [7], [25], [26]. Other concomitant drugs, such as corticosteroids and non-steroidal anti-inflammatories (NSAIDs) were allowed during the study.

### Outcome definition

#### Non-response

MTX clinical response was recorded at time of each visit. Non-response was defined when patients presented a DAS28>3.2, calculated and defined as described by Prevoo *et al.* [27], in two consecutive evaluations. Therefore, non-response to MTX had a minimum period of MTX therapy, at least, of six months.

#### Toxicity

The occurrence of MTX-related toxicity, defined when patients presented any adverse drug reaction (ADR) related to MTX, was recorded upon each visit. The type of ADR was classified in System Organ Class (SOC) disorders, in accordance with Common Terminology Criteria for Adverse Events (CTCAE) [28].

### Samples handling and *TYMS* genotyping

Whole blood samples from each patient were obtained with standard venipuncture technique in ethylenediaminetetraacetic acid (EDTA) containing tubes. Genomic DNA was extracted with QIAamp DNA Blood Mini Kit (QIAGEN, Hilden, Germany) according to manufacturer instructions and total genomic DNA was quantified, and its purity analyzed, using the NanoDrop 1000 Spectrophotometer v3.7 (Thermo Scientific, Wilmington DE, USA).

#### TSER polymorphisms

28 bp VNTR polymorphism (rs34743033) and SNP C>G (rs2853542) at the twelfth nucleotide of the second repeat of 3R allele were genotyped as described by Lima *et al.* [29]. For quality control, 10% of the samples were randomly selected for a second analysis and 10% percent of cases were confirmed by automated sequencing in a 3130×l Genetic Analyzer using the Kit BigDye Terminator v3.1 (Life Technologies, Foster City, CA, USA). Results were 100% concordant.

#### TYMS 1494del6 polymorphism

1494del6 polymorphism (rs34489327) was genotyped as described by Lima *et al.* [29] with slight modifications. PCR products were purified with USB ExoSAP-IT (Affymetrix, Santa Clara, CA, USA) before cycle sequencing. Sequence reactions were carried out using the sequencing Kit BigDye Terminator v.3.1 (Life Technologies, Foster City, CA, USA) according to manufacturer's specifications. The sequencing profile was 30 cycles at 96°C for 10 seconds, 55°C for 10 seconds and 60° for 60 seconds, followed by an extension cycle at 60°C for 10 minutes. The sequence products were purified with illustra Sephadex G-50 Fine DNA Grade (GE Healthcare, Fairfield, CT, EUA) columns, denatured with Hi-Di Formamide and run in an 3130×l Genetic Analyzer (Life Technologies, Foster City, CA, USA). For quality control, 10% of the samples were randomly selected for a second analysis and results were 100% concordant.

### Polymorphisms classification and linkage disequilibrium measure

TSER polymorphisms were classified according to their theoretical TS functional *status* as previously described [6] and grouped by predicted expression levels, as follow: low expression genotypes (2R2R, 2R3RC and 3RC3RC), median expression genotypes (2R3RG and 3RC3RG) and high expression genotype (3RG3RG). Haplotype analysis was performed using a two-stage iterative method named expectation maximization algorithm (SNPStats software) [30]. In order to estimate LD between pairs of alleles at TSER and *TYMS* 1494del6 *loci*, *D′* coefficients were calculated in Arlequin for Windows, Version 3.11 (University of Berne, Bern, Switzerland) [31] with 100,000 number of steps in Markov chain. The measure was interpretable as the proportion of maximum possible level of association between two *loci*, given the allele frequencies, ranging from 0 (linkage equilibrium) to 1 (complete LD) [32]. Possible haplotypes were tested for association with risk for non-response to MTX and for MTX-related toxicity by taking the most frequent haplotype as reference.

### Statistical analysis

Statistical analyses were performed with either IBM SPSS Statistics for Windows, Version 20.0 (IBM Corp, Armonk, NY, USA), OpenEpi for Windows, Version 2.3.1 [33] and SNPStats software [30]. Genotype and allele frequencies were assessed and tested for Hardy-Weinberg equilibrium (HWE). All statistical tests were two-sided and a probability (*p*) value of 5% or less was considered as statistically significant. The Pearson Chi-square test or Fisher's exact test were used to compare the outcome variables and *TYMS* polymorphisms. The odds ratio (OR) and the correspondent 95% confidence intervals (CI) were calculated as a measure of the association between the categorical variables. To correct for multiple comparisons, Bonferroni's method was applied in order to control the false positive rate, and a significance level of α = 0.05/(n comparisons) was used [34]. Forest plot was performed using MedCalc software for Windows, Version 13.1.2 [35]. Multivariate analysis with binary logistic regression was used to identify which *TYMS* genotypes or haplotypes could predict the occurrence of non-response to MTX and MTX-related toxicity. This analysis was performed adjusting to potential confounding clinicopathological variables in three steps. In the first step patient-related variables (age, gender and smoking) were considered; in a second step, beyond patient-related variables, disease-related variables (diagnosis age and disease duration) were added; and in a third step, beyond patient and disease-related variables, treatment-related variables (folic acid supplementation, corticosteroids therapy, use of NSAIDs, other concomitant DMARDs used and MTX administration characteristics - dose, treatment duration and administration route) were also considered.

## Results

### Population description

This study included follow-up data of 233 patients, 196 (84.1%) females and 37 (15.9%) males, with a mean age of 51±11.6 years old, of which 32 (13.7%) were smokers. Considering the disease-related variables, the mean age at diagnosis was 40.3±13.2 years old and the median disease duration was 7.0 years (0.3–51.0). All 233 (100.0%) patients were treated with MTX with a median dose of 15.0 mg/week (2.5–25.0), 118 (50.6%) complied regularly to folic acid supplementation, 188 (80.7%) were under corticosteroid therapy and 170 (73.0%) used NSAIDs.

Non-response to MTX (DAS28 >3.2 in two consecutive evaluations) was observed in 128 (54.9%) patients. Regarding disease activity, the mean for DAS28 was 4.2±1.3. MTX-related toxicity was registered in 77 (33.0%) patients. The observed ADRs were classified in SOCs disorders as follow: 58 (75.3%) gastrointestinal disorders (abdominal distension, diarrhea, dyspepsia, nauseas, stomach pain and/or vomiting); 9 (11.7%) skin and subcutaneous tissue disorders (alopecia, rash maculo-papular and rheumatoid nodulosis exacerbation); 5 (6.5%) hepatobiliary disorders (determined by transaminases serum elevation); and 5 (6.5%) respiratory, thoracic and mediastinal disorders (hypersensitivity pneumonitis). Since the number of cases in each SOCs disorders were small, the evaluation of *TYMS* polymorphisms with clinical relevance as possible biomarkers of MTX-related toxicity was performed for MTX-related overall toxicity.

### *TYMS* genotype and haplotype analyses

Genotypes distribution of *TYMS* polymorphisms was in HWE (*p*>0.050) in the studied population. Frequencies of 28 bp VNTR alleles and genotypes were: 2R allele 41.8%; 3R allele 57.3%; 4R allele 0.9%; 2R2R 15.0% (n = 35); 2R3R 53.7% (n = 125); 3R3R 29.6% (n = 69); and 3R4R 1.7% (n = 4). Due to the low frequency of 3R4R genotype, it was excluded from the analyses. Considering TSER polymorphisms, genotypes distribution was: 2R allele 42.6%; 3RC allele 33.0%; 3RG allele 24.4%; 2R2R 15.3% (n = 35); 2R3RC 29.7% (n = 68); 2R3RG 24.9% (n = 57); 3RC3RC 12.6% (n = 29); 3RC3RG 10.9% (n = 25); and 3RG3RG 6.6% (n = 15). According to TS theoretical functional *status*, genotypes frequencies were: low expression 57.6% (n = 132); median expression 35.8% (n = 82); and high expression 6.6% (n = 15). Frequencies of 1494del6 alleles and genotypes were: 6pb+ allele 70.0%; 6bp− allele 30.0%; 6bp+6bp+ 48.9% (n = 114); 6bp+6bp− 41.6% (n = 97); and 6bp−6bp− 9.5% (n = 22).

Haplotype analysis revealed that 28 bp VNTR and 1494del6 polymorphisms were in LD (*p*<0.00001). Alleles 2R and 6bp+, and alleles 3R and 6bp− were the most linked ones (*D′* = 0.67 for both). The analysis demonstrated four haplotypes: 2R6bp+ 38.4%; 2R6bp− 4.1%; 3R6bp+ 31.7% and 3R6bp− 25.8%. TSER and 1494del6 polymorphisms were also in LD (*p*<0.00001). Alleles 2R and 6bp+ (*D′* = 0.67) and 3RG and 6bp− (*D′* = 0.48) demonstrated to be the most linked ones. This analysis showed six haplotypes: 2R6bp+ 38.4%; 2R6bp− 4.1%; 3RC6bp+ 22.7%; 3RG6bp+ 9.0%; 3RC6bp− 10.3%; and 3RG6bp− 15.5%.

### *TYMS* genotypes and MTX therapeutic outcome

Table 1 reports the relation between *TYMS* polymorphisms and MTX therapeutic outcome both regarding MTX non-response and toxicity.

**Table 1:** *Thymidylate synthase* polymorphisms and methotrexate therapeutic outcome.

	MTX Response				MTX Toxicity			
	Response	Non-Response	*p*	OR (95% CI)	Non-Toxicity	Toxicity	*p*	OR (95% CI)
***TYMS*** ** 28 bp VNTR (rs34743033)** #								
2R2R	19 (54.3)	16 (45.7)		Reference	25 (71.4)	10 (28.6)		Reference
2R3R	62 (49.6)	63 (50.4)	0.624	1.21 (0.57–2.56)	78 (62.4)	47 (37.6)	0.324	1.51 (0.67–3.41)
3R3R	21 (30.4)	48 (69.6)	**0.018** ¥	2.71 (1.17–6.29)	50 (72.5)	19 (27.5)	0.911	0.95 (0.39–2.35)
2R carriers	81 (50.6)	79 (49.4)		Reference	103 (64.4)	57 (35.6)		Reference
3R3R	21 (30.4)	48 (69.6)	**0.005** ¥	2.34 (1.29–4.27)	50 (72.5)	19 (27.5)	0.233	0.69 (0.37–1.28)
2R2R	19 (54.3)	16 (45.7)		Reference	25 (71.4)	10 (28.6)		Reference
3R carriers	83 (42.8)	111 (57.2)	0.208	1.59 (0.77–3.27)	128 (66.0)	66 (34.0)	0.529	1.29 (0.58–2.84)
2R allele	100 (51.3)	95 (48.7)		Reference	128 (65.6)	67 (34.4)		Reference
3R allele	104 (39.5)	159 (60.5)	**0.012** ¥	1.61 (1.09–2.38)	178 (67.7)	85 (32.3)	0.647	0.91 (0.60–1.38)
**TSER polymorphisms (rs2853542** * **and rs34743033)**								
*Functional 2R*								
2R2R	19 (54.3)	16 (45.7)		Reference	25 (71.4)	10 (28.6)		Reference
2R3RC	32 (47.1)	36 (52.9)	0.487	1.34 (0.59–3.03)	44 (64.7)	24 (35.3)	0.492	1.36 (0.56–3.31)
3RC3RC	9 (31.0)	20 (69.0)	0.062	2.64 (0.94–7.39)	23 (79.3)	6 (20.7)	0.469	0.65 (0.20–2.08)
*Functional 3R*								
2R3RG	30 (52.6)	27 (47.4)		Reference	34 (59.6)	23 (40.4)		Reference
3RC3RG	6 (24.0)	19 (76.0)	**0.016** ¥	3.52 (1.23–10.10)	16 (64.0)	9 (36.0)	0.710	0.83 (0.31–2.20)
3RG3RG	6 (40.0)	9 (60.0)	0.384	1.67 (0.52–5.30)	11 (73.3)	4 (26.7)	0.384§	0.54 (0.15–1.90)
2R allele	100 (51.3)	95 (48.7)		Reference	128 (65.6)	67 (34.4)		Reference
3RC allele	56 (37.1)	95 (62.9)	**0.008** ¥	1.79 (1.13–2.82)	106 (70.2)	45 (29.8)	0.369	0.81 (0.50–1.31)
3RG allele	48 (42.9)	64 (57.1)	0.155	1.40 (0.86–2.30)	72 (64.3)	40 (35.7)	0.810	1.06 (0.63–1.78)
**TSER polymorphisms grouped according to theoretically TS expression levels** **								
Low expression	60 (45.5)	72 (54.5)		Reference	92 (69.7)	40 (30.3)		Reference
Median expression	36 (43.9)	46 (56.1)	0.824	1.07 (0.61–1.85)	50 (61.0)	32 (39.0)	0.189	1.47 (0.83–2.63)
High expression	6 (40.0)	9 (60.0)	0.687	1.25 (0.42–3.71)	11 (73.3)	4 (26.7)	1.000§	0.84 (0.25–2.79)
Low+Median expression	96 (44.9)	118 (55.1)		Reference	142 (66.4)	72 (33.6)		Reference
High expression	6 (40.0)	9 (60.0)	0.714	1.22 (0.42–3.55)	11 (73.3)	4 (26.7)	0.778§	0.72 (0.22–2.33)
Low expression	60 (45.5.)	72 (54.5)		Reference	92 (69.7)	40 (30.3)		Reference
Median+High expression	42 (43.4)	55 (56.7)	0.746	1.09 (0.64–1.85)	61 (62.9)	36 (37.1)	0.279	1.36 (0.78–2.36)
***TYMS*** ** 1494del6 (rs34489327)**								
6bp+6bp+	61 (53.5)	53 (46.5)		Reference	78 (68.4)	36 (31.6)		Reference
6bp+6bp−	38 (39.2)	59 (60.8)	**0.038**	1.79 (1.03–3.10)	59 (60.8)	38 (39.2)	0.249	1.40 (0.79–2.46)
6bp−6bp−	6 (27.3)	16 (72.7)	**0.024** ¥	3.07 (1.12–8.41)	19 (86.4)	3 (13.6)	0.122§	0.34 (0.10–1.23)
6bp+6bp+	61 (53.5)	53 (46.5)		Reference	78 (68.4)	36 (31.6)		Reference
6bp− carriers	44 (37.0)	75 (63.0)	**0.011** ¥	1.96 (1.16–3.31)	78 (65.5)	41 (34.5)	0.641	1.14 (0.66–1.97)
6bp+ carriers	99 (46.9)	112 (53.1)		Reference	137 (64.9)	74 (35.1)		Reference
6bp−6bp−	6 (27.3)	16 (72.7)	0.078	2.36 (0.89–6.26)	19 (86.4)	3 (13.6)	0.055§	0.29 (0.08–1.02)
6bp+ allele	160 (49.2)	165 (50.8)		Reference	215 (66.2)	110 (33.8)		Reference
6bp− allele	50 (35.5)	91 (64.5)	**0.006** ¥	1.76 (1.15–2.71)	97 (68.8)	44 (31.2)	0.578	0.89 (0.57–1.38)

Results are expressed in n (%). *p* value<0.05 was considered to be of statistical significance (highlighted in bold).

§ Fisher's exact test used when number of cases of one cell was less than 5.

¥ Statistically significant when *p* values were adjusted for multiple comparisons correction using Bonferroni's method (α = 0.05/n comparisons).

# 3R4R genotype (n = 4) was excluded from analyses due to the low frequency.

*rs2853542 - *TYMS* SNP C>G on 3R allele.

**Genotypes theoretically associated with TS expression: a) high: 3RG3RG; b) median: 2R3RG and 3RC3RG; c) low: 2R2R, 2R3RC and 3RC3RC.

bp: base pairs; C: cytosine; del: deletion; G: guanine; OR: odds ratio; R: repeat; SNP: single nucleotide polymorphism; TS: thymidylate synthase (protein); TSER: *TYMS* enhancer region; *TYMS*: thymidylate synthase (gene); VNTR: variable number tandem repeat.

#### Non-response

In relation to 28 bp VNTR polymorphism, 3R allele was significantly associated with non-response to MTX when compared to 2R allele (*p* = 0.012, OR = 1.61). In addition, 3R homozygotes were associated with more than 2-fold increased risk for non-response to MTX when compared to 2R homozygotes (*p* = 0.018, OR = 2.71) and 2R carriers (*p* = 0.005, OR = 2.34) and remained significant after corrected for multiple comparisons. For TSER polymorphisms, 3RC allele shown to be associated with non-response to MTX when compared to 2R allele (*p* = 0.008, OR = 1.79). Furthermore, and attending to functional 3R, 3RC3RG was related with more than 3-fold increased risk for non-response to MTX when compared to 2R3RG (*p* = 0.016, OR = 3.52), which remained significant after multiple comparisons correction. Considering the 1494del6 polymorphism, 6bp− allele was significantly associated with non-response to MTX when compared to 6bp+ allele (*p* = 0.006, OR = 1.76). Moreover, and compared to 6bp+ homozygotes, 6bp+6bp− (*p* = 0.038, OR = 1.79), 6bp−6bp− (*p* = 0.024, OR = 3.07) and 6bp− carriers (*p* = 0.011, OR = 1.96) presented a statistically significant increased risk for non-response to MTX and, excepting for 6bp+6bp−, continued significant after correcting for multiple comparisons.

#### Toxicity

No statistically significant differences were observed in relation to *TYMS* genotypes and MTX-related overall toxicity.

### *TYMS* haplotypes and MTX therapeutic outcome

Table 2 represents the relationship between *TYMS* haplotypes and MTX therapeutic outcome both regarding MTX non-response and toxicity.

**Table 2:** *Thymidylate synthase* haplotypes and methotrexate therapeutic outcome.

	MTX Response				MTX Toxicity			
*TYMS* Haplotypes	Response	Non-Response	*p*	OR (95% CI)	Non-Toxicity	Toxicity	*p*	OR (95% CI)
**Based on ** ***TYMS*** ** 28 bp VNTR and ** ***TYMS*** ** 1494del6 polymorphisms**								
2R6bp+	43.0	30.0		Reference	36.2	33.9		Reference
2R6bp−	6.0	7.4	0.360	1.70 (0.54–5.32)	5.7	10.2	0.190	2.20 (0.69–7.03)
3R6bp+	33.9	34.5	0.100	1.55 (0.92–2.60)	33.1	37.8	0.490	1.23 (0.69–2.20)
3R6bp−	17.1	28.1	**0.001**	2.54 (1.46–4.43)	25.0	18.1	0.320	0.74 (0.41–1.34)
**Based on TSER and ** ***TYMS*** ** 1494del6 polymorphisms**								
2R6bp+	43.2	30.2		Reference	36.2	34.8		Reference
2R6bp−	5.8	7.2	0.360	1.70 (0.55–5.24)	5.6	9.3	0.220	2.02 (0.66–6.20)
3RC6bp+	21.2	25.6	**0.041**	1.79 (1.03–3.12)	23.8	23.6	0.820	1.07 (0.59–1.95)
3RC6bp−	6.2	11.8	**0.013**	2.80 (1.25–6.25)	10.9	6.0	0.240	0.55(0.21–1.47)
3RG6bp+	12.5	8.7	0.880	1.06 (0.50–2.24)	9.3	13.3	0.300	1.53 (0.69–3.38)
3RG6bp−	11.1	16.5	**0.009**	2.39 (1.24–4.59)	14.2	13.0	0.810	0.92 (0.46–1.82)

Results are expressed in estimated frequencies (%) under linkage disequilibrium. *p* value<0.05 was considered to be of statistical significance (highlighted in bold).

bp: base pairs; C: cytosine; del: deletion; G: guanine; OR: odds ratio; R: repeat; TSER: *TYMS* enhancer region; *TYMS*: thymidylate synthase (gene); VNTR: variable number tandem repeat.

#### Non-response

3R6bp− haplotype was found significantly associated with non-response to MTX when compared to 2R6bp+ haplotype (*p* = 0.001, OR = 2.54). Moreover, 3RC6bp+, 3RC6bp− and 3RG6bp− haplotypes were statistically significant associated with non-response to MTX when compared to 2R6bp+ haplotype (*p* = 0.041, OR = 1.79; *p* = 0.013, OR = 2.80; and *p* = 0.009, OR = 2.39, respectively).

#### Toxicity

No statistically significant differences were observed in relation to *TYMS* haplotypes and MTX-related overall toxicity.

### Multivariate analysis

**Figure 2:**
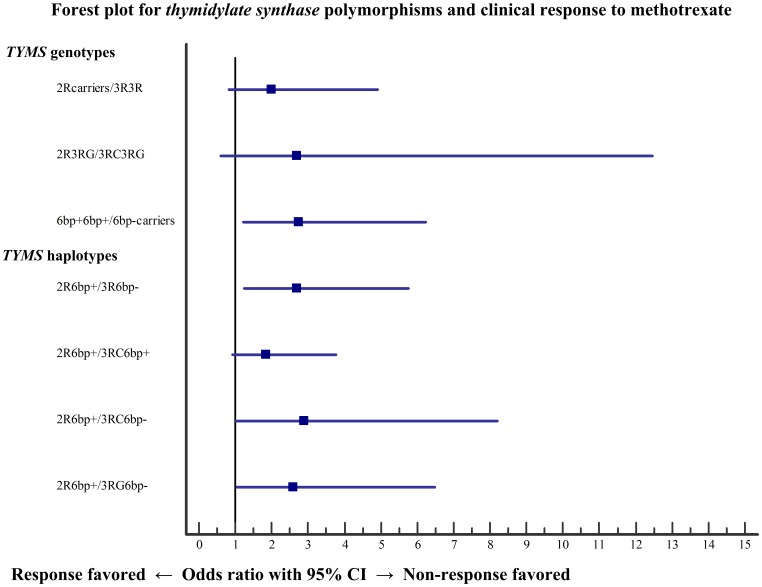
Forest plot of multivariate analysis in the association of *thymidylate synthase* genotypes and haplotypes with clinical response to methotrexate. Odds ratio and 95% confidence intervals are reported for clinical response to methotrexate. bp: base pairs; C: cytosine; CI: confidence interval; del: deletion; G: guanine; R: repeat; TYMS: thymidylate synthase (gene).

Multivariate analysis was performed in three steps adjusting to potential confounding variables. Table 3 shows multivariate analysis results of *TYMS* genotypes and haplotypes and clinical response to MTX. Figure 2 resumes the impact of all potential confounding variables in the association of *TYMS* genotypes and haplotypes with clinical response to MTX. Regarding *TYMS* genotypes, results demonstrated that 6bp− carriers were statistically significant associated with more than 2-fold increased risk for non-response to MTX when compared to 6bp+ homozygotes (*p* = 0.016, OR = 2.74). According to *TYMS* haplotypes, our results shown that haplotypes harboring simultaneously 3R and 6bp− alleles were statistically significant associated with almost 3-fold increased risk for non-response to MTX when compared to 2R6bp+ haplotype.

**Table 3:** Multivariate analysis of *thymidylate synthase* polymorphisms and clinical response to methotrexate.

	Patient-related		Patient-+Disease-related		Patient-+Disease-+Treatment-related	
	*p*	OR (95% CI)	*p*	OR (95% CI)	*p*	OR (95% CI)
***TYMS*** ** genotypes**						
***TYMS*** ** 28 bp VNTR (rs34743033)**						
2R carriers		Reference		Reference		Reference
3R3R	**0.013**	2.23 (1.19–4.17)	**0.013**	2.24 (1.19–4.21)	0.135	1.99 (0.81–4.91)
**TSER polymorphisms**						
*Functional 3R*						
2R3RG		Reference		Reference		Reference
3RC3RG	0.069	2.90 (0.92–9.13)	0.071	2.91 (0.91–9.25)	0.203	2.70 (0.59–12.47)
***TYMS*** ** 1494del6 (rs34489327)**						
6bp+6bp+		Reference		Reference		Reference
6bp− carriers	**0.003**	2.33 (1.32–4.10)	**0.003**	2.38 (1.34–4.23)	**0.016**	2.74 (1.21–6.23)
***TYMS*** ** haplotypes**						
**Based on ** ***TYMS*** ** 28 bp VNTR and ** ***TYMS*** ** 1494del6 polymorphisms**						
2R6bp+		Reference		Reference		Reference
3R6bp−	**<0.001**	2.87 (1.59–5.19)	**<0.001**	2.92 (1.60–5.32)	**0.012**	2.68 (1.25–5.75)
**Based on TSER and ** ***TYMS*** ** 1494del6 polymorphisms**						
2R6bp+		Reference		Reference		Reference
3RC6bp+	**0.041**	1.81 (1.03–3.20)	**0.035**	1.86 (1.05–3.31)	0.090	1.85 (0.91–3.76)
3RC6bp−	**0.012**	2.97 (1.28–6.93)	**0.018**	2.75 (1.19–6.32)	**0.048**	2.89 (1.01–8.21)
3RG6bp−	**0.004**	2.78 (1.39–5.56)	**0.003**	3.06 (1.49–6.31)	**0.043**	2.60 (1.04–6.49)

*P* value<0.05 is considered to be of statistical significance (highlighted in bold).

Adjusted variables include: 1) patient-related variables (age, gender and smoking); 2) disease-related variables (diagnosis age and disease duration); and 3) treatment-related variables (folic acid supplementation, corticosteroids, non-steroidal anti-inflammatories, other concomitant disease-modifying antirheumatic drugs and methotrexate administration characteristics - dose, treatment duration and administration route). Genetic variables include: *TYMS* genotypes and *TYMS* haplotypes.

bp: base pairs; C: cytosine; del: deletion; G: guanine; OR: odds ratio; R: repeat; TSER: *TYMS* enhancer region; *TYMS*: thymidylate synthase (gene); VNTR: variable number tandem repeat.

## Discussion

Thymidylate synthase is a key enzyme for DNA synthesis and repair [11], [12] inhibited by MTXPGs and, therefore, contributes for MTX antiproliferative and anti-inflammatory effects [10]. In fact, TS levels were found to be predictive of MTX therapeutic outcome [13], [14]. Since genetic polymorphisms in *TYMS* have been associated with TS levels [6], in this study we aimed to elucidate the influence of *TYMS* polymorphisms (28 bp VNTR, SNP C>G and 1494del6) in MTX therapeutic outcome of Portuguese RA patients.

All patients enrolled in this study were recruited within a well-defined geographical area and were of Caucasian ethnicity, with gender and age at time of diagnosis distributions similar to other reported populations [36], [37]. Genotypes distribution of *TYMS* polymorphisms was in HWE and was similar to those found for other Caucasian populations [21], [38], [39]. Nevertheless, and despite the potential of our results, possible study limitations include: 1) relatively reduced population size; 2) presence of other *TYMS* polymorphisms that possibly could alter TS expression or functionality; 3) limited screen of some important genes that codify other enzymes involved in MTX action mechanism.

### *TYMS* genotypes and MTX therapeutic outcome

#### Non-response

Among our population and regarding 28 bp VNTR polymorphism, 3R allele was associated with risk for non-response to MTX, which increases in the presence of both 3R alleles, in accordance to previous studies [13], [40]. Literature describes 3R allele as associated with higher TS levels [19], [20] and TS levels as predictive of clinical response to MTX [13], [40]. Moreover, 3R allele has been associated with higher MTX doses required [13] and higher RA disease activity [40]. Despite the significant univariate analysis results, multivariate analysis did not confirm them. Additionally, other studies demonstrated associations between 3R homozygotes and response to MTX [39] or showed no association [21], [41]–[43]. It has been suggested by some authors that it is of greater importance to consider the SNP C>G on 3R allele and analyze the TSER polymorphisms instead of studying 28 bp VNTR polymorphism alone. 3RG allele was associated with higher transcriptional activity and translation efficiency due to its increased ability to complex with the USF protein [15], [44]. Accordingly, the number of functional E-box in both 2R and 3RC alleles should be the same [6], [15], which should reveal that patients with these genotypes would have similar TS expression and, consequently, a resembling clinical response. However, our results seem to demonstrate that 2R and 3RC alleles are different since 3RC3RG genotype was associated with over 3-fold increased risk for non-response to MTX when compared to 2R3RG. In addition, our results showed that 3RC allele was associated with non-response to MTX, when compared to 2R allele, and 3RC3RC genotype has a non-significant trend for non-response to MTX when compared to 2R2R genotype. Nevertheless, no statistically significant differences were observed attending to TSER polymorphisms grouped according to theoretically TS expression levels and to multivariate analysis. Moreover, a previous study demonstrated that non-response to MTX was associated to 3RG3RG patients [21]. Therefore, the putative relationship between TSER polymorphisms and clinical response to MTX outcome needs further clarification.

In relation to 1494del6 polymorphism, our results demonstrated that 6bp− allele was associated with non-response to MTX. Additionally, multivariate analysis showed that 6bp− carriers were associated with about 3-fold increased risk for non-response to MTX. *In vitro* studies have demonstrated that 6bp− allele has decreased mRNA stability and, thereby reduced TS expression [22], [23], however, in other previously reported studies in RA Caucasian patients no association was observed [41]. Moreover, one study in Psoriasis, a disease where MTX is used in similar doses than RA, 6bp− allele demonstrated a trend for non-response, however, this study included Caucasian and non-Caucasian patients [45]. Studies in Asiatic patients have reported different results, some of them reported an association between 6bp− allele and response [13], [43], while others reported no associations [42], [46]. From all of these results it seems that ethnicity could be an important factor to predict the clinical response to MTX.

#### Toxicity

Regarding the occurrence of MTX-related overall toxicity, our results did not reach significance pertaining to TSER and 1494del6 polymorphisms, in accordance with previously reported studies [21], [41], [42], [46], [47]. Nevertheless, other studies reported significant associations of 28 bp VNTR polymorphism with MTX-related toxicity [38], [48]. To the best of our knowledge this is the first report evaluating the influence of TSER polymorphisms in MTX-related toxicity in RA.

### *TYMS* haplotypes and MTX therapeutic outcome

#### Non-response

Haplotypes may have a particular significance in regard to functionality or as genetic markers for unknown functional variants. Therefore, haplotype analysis was performed, to assess of possible consequences on the phenotype in the copresence of several variants of the same gene. As reported by others [6], [39], [49], [50], *TYMS* polymorphisms were in LD, especially 2R6bp+ and 3RG6bp− haplotypes. Univariate haplotype analysis demonstrated that 3R6bp−, 3RC6bp+, 3RC6bp− and 3RG6bp− haplotypes (haplotypes harboring 3R allele for 28 bp VNTR, 3RC allele for TSER and 6bp− allele for 1494del6) were associated with almost 3-fold increased risk for non-response to MTX. Nevertheless, multivariate analysis showed that haplotypes harboring simultaneously 3R and 6bp− alleles (3R6bp−, 3RC6bp− and 3RG6bp−) were associated with non-response to MTX. This suggests a prominent role of the 3′-UTR polymorphism in predicting the clinical response to MTX and it seems that 6bp− allele can interact differently with 2R and 3R alleles, in agreement with Lurje *et al.* [51]. Additionally, our results suggested that the haplotype revealing more risk for non-response to MTX was 3RC6bp−, which combines the major risk alleles from the 5′UTR (3RC) and from the 3′UTR (6bp−). Only one study in RA has performed haplotype analysis, where an association between 3R6bp− haplotype and response to MTX was demonstrated [39]. Nevertheless, there are some important differences: no reference to SNP C>G; studied population included patients with early RA; and the study evaluated the impact in clinical response to MTX combined therapy with sulfasalazine. Thus, we propose that *TYMS* haplotype analysis should be used in future studies to elucidate the influence of *TYMS* in MTX therapeutic outcome, which could help to interpret these preliminary conflicting data.

#### Toxicity

Regarding MTX-related toxicity, no differences were observed attending to *TYMS* haplotypes. Despite it was expected that *TYMS* haplotypes follow the same tendency as *TYMS* genotypes, to the best of our knowledge no studies analyzed the *TYMS* haplotypes and the development of toxicity arising from MTX in RA.

The observed discrepancies among different studies could be explained by inter-study variability, ethnicity variability, samples sizes, variety of methods used to measure the MTX therapeutic outcome, different treatment regimens, and different genotyping protocols with limited quality of results. Therefore, functional TS studies in RA should be conducted to better understanding TS expression regulation mechanism and its putative importance in establishing more effective clinical therapeutic strategies when MTX is used in RA patients. To the best of our knowledge, this is the first report regarding the study of the association of *TYMS* polymorphisms with MTX therapeutic outcome in Portuguese RA patients. This study concluded that *TYMS* polymorphisms seem to be important to predict clinical response to MTX in RA patients; *TYMS* genotypes and haplotypes harboring 6bp− allele were associated with non-response to MTX; *TYMS* haplotypes harboring simultaneously 3R and 6bp− alleles seem to be predictors of non-response to MTX; and, to elucidate the role of *TYMS* on MTX therapeutic outcome full haplotypic information should be exploited. Despite the potential of our findings, translation into clinical practice requires larger and multicentric studies in order to clearly endorse the utility of these polymorphisms.
